# Increased Risk of Herpes Zoster in Diabetic Patients Comorbid with Coronary Artery Disease and Microvascular Disorders: A Population-Based Study in Taiwan

**DOI:** 10.1371/journal.pone.0146750

**Published:** 2016-01-11

**Authors:** Chi-Chen Ke, Hui-Chin Lai, Ching-Heng Lin, Chih-Jen Hung, Der-Yuan Chen, Wayne H-H. Sheu, Ping-Wing Lui

**Affiliations:** 1 Department of Anesthesiology, Taichung Veterans General Hospital, Taichung, Taiwan; 2 Department of Medical Research, Taichung Veterans General Hospital, Taichung, Taiwan; 3 Division of Allergy, Immunology and Rheumatology, Department of Internal Medicine, Taichung Veterans General Hospital, Taichung, Taiwan; 4 Division of Endocrinology and Metabolism, Department of Internal Medicine, Taichung Veterans General Hospital, Taichung, Taiwan; 5 Institute of Pharmacology, National Yang Ming University, Taipei, Taiwan; University of California Riverside, UNITED STATES

## Abstract

We investigated the association between the risk of herpes zoster (HZ) and diabetes-related macrovascular comorbidities and microvascular disorders in diabetic patients. This retrospective study included 25,345 patients with newly identified HZ and age- and gender-matched controls retrieved from the National Health Insurance Research Database in Taiwan during the period of 2005 to 2011. Multivariate logistic regression analyses were used to calculate the odds ratios (OR) and to assess the risk factors for HZ in diabetic patients with associated macrovascular or microvascular disorders. Risk factors for HZ were significantly increased in cases of diabetes mellitus (DM) compared with those in cases of non-DM controls (20.2% vs. 17.0%, OR = 1.24, p<0.001). Results of age- and gender-adjusted analyses demonstrated a significantly higher risk of HZ in DM patients with accompanying coronary artery disease (CAD) (adjusted OR = 1.21, p<0.001) and microvascular disorders (aOR = 1.32, p<0.001) than in DM patients with other comorbidities but no microvascular disorders. Patients who took thiazolidinedione, alpha-glucosidase inhibitors and insulin had a higher HZ risk than those taking metformin or sulphonylureas alone (aOR = 1.11, 1.14 and 1.18, p<0.001, respectively). Patients who took insulin alone or in combination with other antidiabetic agents had a significantly higher risk of HZ (aOR = 1.25, p<0.001) than those who received monotherapy. Diabetic patients comorbid with coronary artery disease and associated microvascular disorders had an increased risk of HZ occurrence.

## Introduction

Herpes zoster (HZ) is caused by the reactivation of varicella-zoster virus (VZV) latent in the sensory ganglia after primary infection. It is a painful blister or rash on the afflicted dermatomes secondary to the spreading of the virus along the sensory nerve fibers [1–3]. The annual incidence rate of HZ is similar to that in North America, Europe and the Asia-Pacific region, ranging from 3~5 cases per 1000 person-years (PY). The incidence of HZ substantially increases with age and immunosuppression, affecting more than 50% of subjects aged >85 years [4].

Diabetic patients are susceptible to HZ secondary to VZV reactivation as cell-mediated immunity (CMI) declines during the process [5]. Diabetes mellitus (DM) is commonly regarded as a prothrombotic disorder associated with altered innate or adaptive immunity and endothelium dysfunction secondary to inflammation [6]. Thus, patients with DM have accompanying chronic comorbid diseases or related vascular complications, which are considered a major preventable risk factor for cardiovascular diseases. Diabetic patients are 2 to 4 times more likely to develop cardiovascular or cerebrovascular diseases than non-diabetic patients [7–9]. When patients have two or more coexisting comorbidities, there is an increased risk of HZ occurrence [10]. On the other hand, a retrospective cohort study in the UK demonstrated that HZ was an independent risk factor for VZV vasculopathy such as stroke, transient ischemic attack and myocardial infarction [11]. However, few studies have explored the association between the risk of HZ in diabetic patients comorbid with coronary artery diseases (CAD) and diabetes-related microvascular disorders.

The objectives of this study were to determine whether the risk of HZ occurrence would be escalated in diabetic patients comorbid with coronary artery diseases or in combination with other related microvascular disorders. In addition, using data retrieved from a nationwide database in Taiwan, we assessed the impact of anti-diabetic agents on the risk of HZ in these patients.

## Materials and Methods

### Data source

Since 1995, the National Health Insurance program has provided comprehensive medical coverage to 99% of the population of 23 million in Taiwan. The National Health Research Institutes (NHRI) have established a National Health Insurance Research Database (NHIRD). All medical records, including demographic characteristics, underlying comorbidities, as well as the date of the first outpatient visit, inpatient admission and discharge, and disease status at discharge, are made available in anonymous format for research purposes. The NHIRD also provides information about inpatient and outpatient use of medical services for all beneficiaries. Patients diagnosed with HZ were identified through claims data of the NHIRD by the International Classification of Diseases, 9^th^ Revision, Clinical Modification (ICD-9-CM) codes, which were used to define diseases. As the NHRI made the claim data available in an anonymous format which provided the individuals cannot be identified individually, the retrospective studies do not need the ethical approval from ethics committees in Taiwan.

### Collection of cases and controls

**Fig 1:**
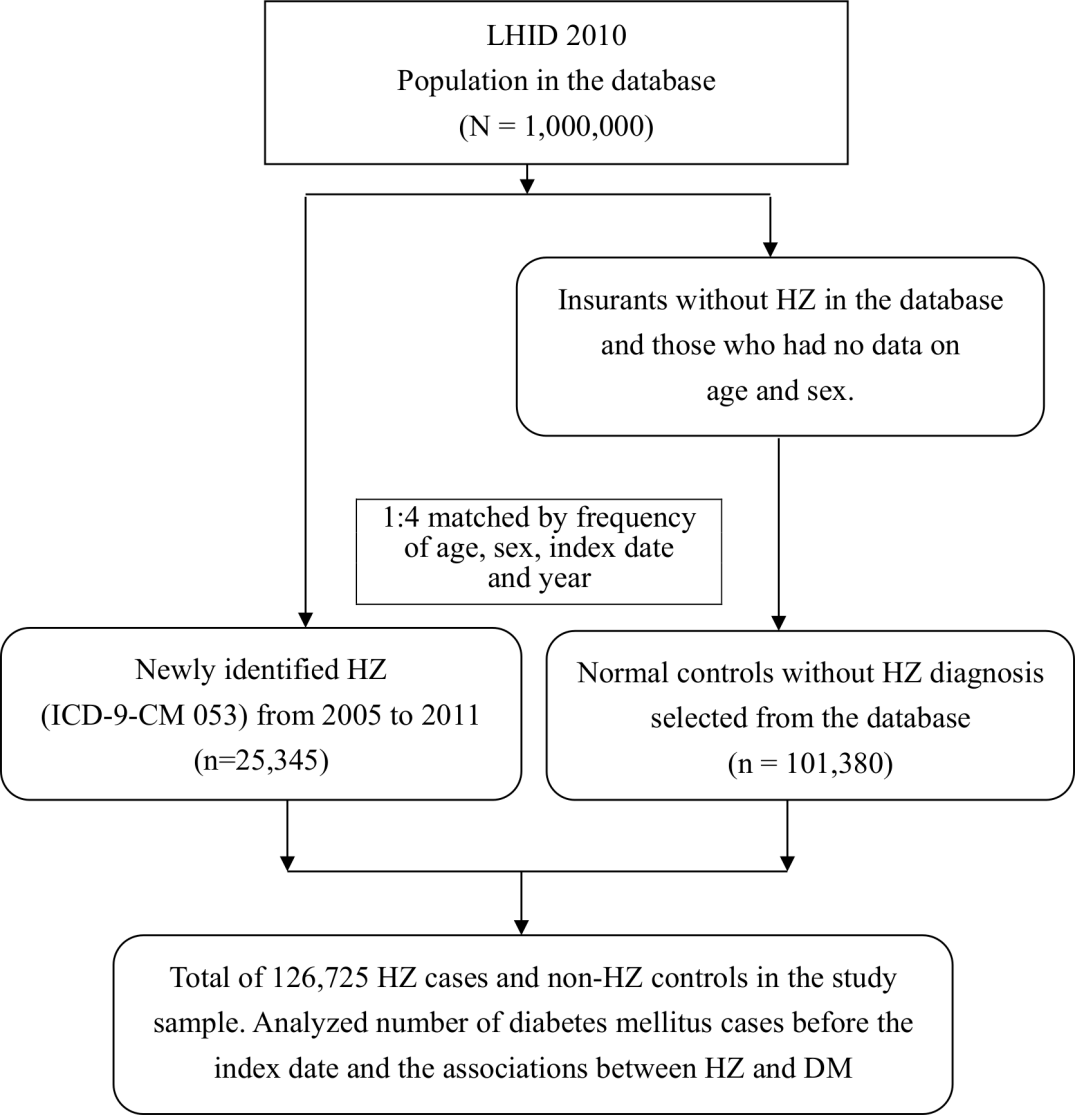
Flowchart for data retrieval of patients diagnosed with herpes zoster (HZ) and controls from the National Health Insurance Research Database in Taiwan from 2005 to 2011. LHID = Longitudinal Health Insurance Database.

In this population-based retrospective case control study, we used a systemic sampling of patient data from a total of 1,000,000 subjects, which was released by the NHIRD (Fig 1). Data on a total of 25,345 patients aged ≥18 years with newly identified HZ (ICD-9-CM code 053) were retrieved from the claims database during the period of 2005 to 2011. The inclusion criteria for data retrieval were: patients with the principal diagnosis of HZ who visited an outpatient department at least twice or those who were admitted to the hospital at least once. We matched HZ patients and non-HZ control subjects 1:4 by frequency of age, gender, and index date. The index date was the date on which herpes zoster was diagnosed. Non-herpes zoster subjects were selected as controls from the index date for matching with HZ patients.

Coexisting medical illnesses were identified as potential confounders by ICD-9-CM diagnostic codes, including diabetes mellitus (ICD-9-CM code 250), stroke (ICD-9-CM 430–438), coronary artery disease (ICD-9-CM 414), chronic kidney disease (ICD-9-CM code 585), diabetes with peripheral circulatory disorders (ICD-9-CM 250.7), diabetes with renal manifestations (ICD-9-CM 250.4), diabetes with ophthalmic manifestations (ICD-9-CM 250.5), and diabetes with neurological manifestations (250.6). Furthermore, we also recorded information about prescribed anti-diabetic agents according to the anatomic therapeutic chemical [ATC] classification system. Drugs included thiazolidinedione (TZD): rosiglitazone (A10BG02), pioglitazone (A10BG03) and metformin (A10BA02), as well as sulfonylureas (A10BB), alpha glucosidase inhibitors (A10BF) and insulin (A10A).

### Statistical analysis

Differences between the HZ group and non-HZ group were compared using a Chi-square test based on the social and demographic characteristics as well as underlying comorbidities. A univariate logistic regression model was used to compare the risk of HZ with baseline characteristics as shown by odds ratio (OR) with a 95% confidence interval (CI). In addition, multivariate logistic regression was performed to estimate the associations among complications, comorbidities, drugs used, DM duration and the risk of HZ infection in DM patients. A two-tailed p value of <0.05 was considered statistically significant. All statistical analyses were performed using the SAS statistical software (version 9.3 for Windows; SAS Institute, Inc., Cary, NC, USA).

## Results

From 2005 to 2011, a total of 25,345 newly diagnosed patients with herpes zoster were enrolled in this study in a 1:4 ratio with control patients without herpes zoster (n = 101,380) (Fig 1). Patients with DM were associated with a 24% increase in the risk of HZ as compared to those without diabetes (OR = 1.24, 95% CI = 1.19–1.28, p<0.001, Table 1). The average incidence of HZ from 2005~2011 was estimated at 3.6 cases per 1000 PY, with the highest rate in patients >70 years old (9.53 cases per 1000 PY), and the lowest rate in those <30 years old (1.53 cases per 1000 PY). Age-stratified analysis revealed a higher risk for HZ in all age groups with the highest risk in patients <30 years old (OR = 1.54, 95% CI = 1.03–2.30, p<0.05) and the lowest risk in those >70 years old (OR = 1.16, 95% CI = 1.09–1.24, p<0.001). Both male and female diabetic patients appeared to have an increased risk of developing herpes zoster compared to those without diabetes, as shown in women (OR = 1.23, 95% CI: 1.17–1.29, p<0.001) and in men (OR = 1.25, 95% CI = 1.19–1.32, p<0.001), respectively.

**Table 1:** Baseline characteristics and number of DM cases among patients with/without herpes zoster, with associations between HZ and DM in a logistic regression model, adjusted for gender and age.

	Herpes Zoster (HZ)								
	No			Yes				Univariate Model	
Variables	n	No. DM	(%)	n	No. DM	(%)	P-value^†^	OR	(95%CI)
**Overall**	101380	17183	(17.0)	25345	5106	(20.2)	<0.001	1.24	(1.19–1.28)***
**Gender**									
Female	54220	9702	(17.9)	13555	2858	(21.1)	<0.001	1.23	(1.17–1.29)***
Male	47160	7481	(15.9)	11790	2248	(19.1)	<0.001	1.25	(1.19–1.32)***
**Age, years**									
Mean ± SD		51.3±19.7			51.9±19.4				
<30	16524	86	(0.5)	4131	33	(0.8)	0.035	1.54	(1.03–2.30)*
30–49	24816	1471	(5.1)	6204	464	(7.5)	<0.001	1.50	(1.34–1.67)***
50–69	40904	9041	(22.1)	10226	2743	(26.8)	<0.001	1.29	(1.23–1.36)***
≧70	19136	6785	(35.5)	4784	1866	(39.0)	<0.001	1.16	(1.09–1.24)***

*p<0.05

**p<0.01

***p<0.001

^†^ p-value of Chi-square test

Interaction p = 0.596 for gender; interaction p<0.001 for age

Results of multivariate logistic regression analysis of the correlation between comorbidities and risk of HZ among diabetic patients are shown in Table 2. After adjusting for age, gender, DM duration and confounding factors, we found that diabetic patients comorbid with coronary artery disease (CAD) alone had a significantly higher risk of developing HZ than patients without CAD (21.2% vs. 18.5%, adjusted OR = 1.21, 95% CI = 1.12–1.31, p<0.001). Regarding the combination of complications associated with the risk of HZ, diabetic patients comorbid with both CAD and microvascular diseases had the highest risk (OR = 1.32, 95% CI = 1.12–1.55, p<0.001), as compared to those without CAD or microvascular diseases.

**Table 2:** Association between comorbidities or diabetes-related complications and risk of herpes zoster among diabetic patients.

	Herpes Zoster (HZ)								
	No (n = 17183)		Yes (n = 5106)						
Variables	n	(%)	n	(%)	P-value^†^	cOR	(95%CI)	aOR	(95%CI)
**Comorbidities**									
Stroke					0.313				
No	14941	(87.0)	4412	(86.4)		1.00	(reference)	1.00	(reference)
Yes	2242	(13.1)	694	(13.4)		1.05	(0.95–1.15)	1.09	(0.99–1.19)
CAD					<0.001				
No	14001	(81.5)	4026	(78.9)		1.00	(reference)	1.00	(reference)
Yes	3182	(18.5)	1080	(21.2)		1.18	(1.09–1.28)***	1.21	(1.12–1.31)***
DM with peripheral circulatory disorders					0.088				
No	16827	(97.9)	4980	(97.5)		1.00	(reference)	1.00	(reference)
Yes	356	(2.1)	126	(2.5)		1.20	(0.97–1.47)	1.21	(0.99–1.49)
DM with nephropathy					0.626				
No	16085	(93.6)	4770	(93.4)		1.00	(reference)	1.00	(reference)
Yes	1098	(6.4)	336	(6.6)		1.03	(0.91–1.17)	1.04	(0.92–1.18)
DM with retinopathy					0.210				
No	16702	(97.2)	4946	(96.9)		1.00	(reference)	1.00	(reference)
Yes	481	(2.8)	160	(3.1)		1.12	(0.94–1.35)	1.13	(0.94–1.35)
DM with neuropathy					0.159				
No	16116	(93.8)	4761	(93.2)		1.00	(reference)	1.00	(reference)
Yes	1067	(6.2)	345	(6.8)		1.09	(0.97–1.24)	1.10	(0.97–1.25)
**Coexisting with diabetes-related complications**^‡^					<0.001				
CAD (-)/microvascular disease (-)	12056	(70.2)	3423	(67.0)		1.00	(reference)	1.00	(reference)
CAD (-)/microvascular disease (+)	1945	(11.3)	603	(11.8)		1.09	(0.99–1.21)	1.10	(1.00–1.22)
CAD (+)/microvascular disease (-)	2575	(15.0)	859	(16.8)		1.18	(1.08–1.28)**	1.21	(1.11–1.32)***
CAD (+)/microvascular disease (+)	607	(3.5)	221	(4.3)		1.28	(1.09–1.50)*	1.32	(1.13–1.55)**

CAD: coronary artery disease; DM: diabetes mellitus; cOR: crude odds ratio; aOR: adjusted odds ratio, adjusted for age, gender, and DM duration

*p<0.05

**p<0.01

***p<0.001

^†^ p-value of Chi-square test

^‡^ Test for a trend cross variable of a combination of complications

Diabetic patients who took anti-diabetic agents such as thiazolidinedione (TZD), alpha-glucosidase inhibitors or insulin had an increased risk of HZ as compared to those who did not take these agents (adjusted OR = 1.11, 1.02 and 1.21, respectively; p<0.01~p<0.001, Table 3). However, there was no significant difference between HZ patients and non-HZ patients who took metformin or sulfonylureas alone. Using logistic regression analysis to determine the risk of HZ in diabetic patients who received different anti-diabetic drugs, we found a significantly higher risk of developing HZ in patients who received insulin therapy or insulin combined with other drugs than those treated with metformin or SU treatment alone (OR = 1.25, 95% CI = 1.11–1.41, p<0.001).

**Table 3:** Association between anti-diabetic agents and risk of herpes zoster among diabetic patients.

	Herpes Zoster (HZ)								
	No (n = 17,183)		Yes (n = 5,106)						
Anti-diabetic Agents	n	(%)	n	(%)	P-value^†^	cOR	(95%CI)	aOR	(95%CI)
Metformin					0.103				
No	8690	(50.6)	2516	(49.3)		1.00	(reference)	1.00	(reference)
Yes	8493	(49.4)	2590	(50.7)		1.05	(0.99–1.12)	1.05	(0.99–1.12)
Sulfonylureas					0.302				
No	9096	(52.9)	2661	(52.1)		1.00	(reference)	1.00	(reference)
Yes	8087	(47.1)	2445	(47.9)		1.03	(0.97–1.10)	1.03	(0.97–1.10)
Thiazolidinedione					0.010				
No	14688	(85.5)	4290	(84.0)		1.00	(reference)	1.00	(reference)
Yes	2495	(14.5)	816	(16.0)		1.12	(1.03–1.22)*	1.11	(1.02–1.21)*
Alpha glucosidase inhibitors					0.005				
No	14753	(85.9)	4303	(84.3)		1.00	(reference)	1.00	(reference)
Yes	2430	(14.1)	803	(15.7)		1.13	(1.04–1.24)**	1.14	(1.04–1.24)**
Insulin					<0.001				
No	13951	(81.2)	4023	(78.8)		1.00	(reference)	1.00	(reference)
Yes	3232	(18.8)	1083	(21.2)		1.16	(1.08–1.26)**	1.18	(1.09–1.27)***
**Anti-diabetic agents in combination**^**‡**^					<0.001				
Metformin only or Sulfonylureas only	1817	(10.6)	488	(9.6)		1.00	(reference)	1.00	(reference)
Metformin + others or Sulfonylureas + others	12134	(70.6)	3535	(69.2)		1.09	(0.98–1.21)	1.08	(0.97–1.20)
Insulin only or Insulin+others	3232	(18.8)	1083	(21.2)		1.25	(1.11–1.41)***	1.25	(1.11–1.42)***

cOR: crude odds ratio; aOR: adjusted odds ratio. Adjusted for age, gender, and DM duration.

*p<0.05

**p<0.01

***p<0.001

^†^ p-value of Chi-square test

^**‡**^ Others include TZD and alpha glucosidase inhibitors

## Discussion

The results of the present study demonstrated that there was an increased risk of HZ in patients comorbid with both DM and CAD as well as diabetes-related microvascular disorders. In addition, diabetic patients who received insulin alone or in combination with other anti-diabetic agents had a significantly higher risk of HZ than those receiving monotherapy.

The incidence of HZ in Taiwan is comparable with that cited in global reports, varying from 1.2~4.9 cases per 1000 PY. In the study by Jih *et al*. [12], the incidence of HZ was 4.89 cases per 1000 PY as compared to 3.62 cases per 1000 PY in the present study. This discrepancy may be attributable to the difference in the inclusion criteria between these studies. Our enrollment criteria were considerably stricter because we required patients to have at least two outpatient visits or one inpatient admission for treatment. In the study by Jih *et al*., patients who had only one outpatient visit or a single inpatient admission were enrolled. Jih *et al*. used a retrospective cohort design, which included inpatient and outpatient claims data, and the authors followed up patients from 2000 to 2006. Our design, however, was a case-control study in which patients were retrospectively assessed from 2005 to 2011 for the presence or absence of HZ infection. Though retrospective cohort and case-control designs have both advantages and disadvantages in answering observational questions, the reliability of both types of studies is similar in providing strength of evidence [13].

Due to a decline in CMI in diabetic patients, the results of our study were in line with those of previous population-based studies, in which diabetic patients were more susceptible to HZ (OR = 1.24) than non-diabetic patients. Guignard *et al*. [5] found that type 2 DM was associated with an enhanced risk of HZ with a hazard ratio of 1.97. In a nested case-control study, Heymann *et al*. also demonstrated an increased risk of HZ with an odds ratio of 1.53 in diabetic patients [14]. However, the result of our study demonstrated a lower risk of HZ in comparison to Guignard’s and Heymann’s studies. Three major factors may contribute to this inconsistency. The first direct factor is the racial differences in the occurrence of herpes zoster among studies conducted in different countries. There are evidences showing that non-white had a significantly lower risk of developing herpes zoster than white [15,16]. The second contributory factor is the difference in health insurance among these countries. Our health insurance is a nationwide healthcare system which included all socioeconomic status in Taiwan with 99% population coverage, which can be a reliable representative of diseases with diagnosis and follow-up. The last critical factor is the difference in age selection. In our study, we only selected adult patients while they included all ages. It is thus possible that if adolescent diabetic patients, mainly Type 1 DM, were not included as occurred in our study, a diminished risk or OR of HZ occurrence was expected. Therefore, the risk of developing HZ in diabetic patients may vary substantially depending on many risk factors, especially in countries where races, population age, weather and health insurance are essentially different.

Similar to the primary immune response to VZV in children, an expansion of antigen-specific memory CD4+ T cells including CD45RA and CD27 cells is triggered in response to the re-exposure of HZ patients to varicella infection. In addition, there was a concomitant change in cytotoxic CD8+ T cells and natural killer cells in the peripheral blood, accompanied by a release of cytokines such as interferon-γ, tumor necrosis factor–α, and interleukin-2 [17]. The imbalance of T cell-mediated immunity in patients predisposed to varicella infection may be attributable to the dysfunction of T lymphocytes involving both VZV-specific memory CD4^+^ and cytotoxic CD8^+^T cells [17, 18]. In association with a compromised CMI, the study by Pedicino *et al*. demonstrated a strong relationship between DM and the imbalance of T-cell homeostasis involving the expansion of CD4^+^CD28^null^ T-cells and the reduction in CD4^+^CD25^+^Foxp3^+^ regulatory T-cells [19].

Patients with cardiovascular diseases such as coronary artery disease (CAD) are associated with a compromised adaptive immune response, not only in CD4^+^T cells but also in CD8^+^T cells [20, 21]. After the first episode of acute coronary syndrome, diabetic patients are usually found to be associated with the expansion of CD4^+^CD28^null^ T-cells, which are manifested as poor glycemic control with worse outcome [22]. In addition, one year after an acute cardiac event, patients with CAD demonstrated an accelerated immunosenescence with an increase in the proportion of CD4^+^CD28^null^ and CD8^+^CD28 ^null^ cells [23]. Joesoef *et al*. reported that the risk of HZ would be increased if patients had coexisting prevalent chronic disorders, such as CAD, DM, hyperlipidemia or hypertension, though most causes of HZ were largely undetermined [10]. Our results further demonstrated that diabetic patients had an escalated risk of HZ (aOR = 1.32) when they were comorbid with CAD or had CAD in combination with other diabetic-related microvascular disorders. There is evidence showing that varicella-zoster virus infection can lead to endothelial dysfunction as the result of increased inflammation with immune-mediated vasculitis [24, 25]. Studies have also demonstrated that patients afflicted with herpes zoster had an increased risk of developing myocardial infarction and stroke as well as other microvascular diseases. It is speculated that when T cell-mediated immunity declined as occurred in diabetic patients, the varicella-zoster virus infection may possibly lead to chronic endothelial inflammation with atherosclerotic plague-formation of the arterial walls. Therefore, it is most likely that the escalated risk of HZ in patients comorbid with both DM and CAD is due to compromised T cell-mediated immunity as well as immune-mediated vasculopathy secondary to varicellar-zoster virus infection. However, our study had an inherent limitation in which the event sequence of DM and CAD could not be identified from our claims database. It is argued that CAD may be an isolated comorbid disorder that accompanies DM or a progressive vascular complication related to diabetes.

Several studies demonstrated an enhanced release of pro-inflammatory cytokines triggered by long-term stress-induced hyperglycemia, which elicited insulin resistance and affected the function of innate immunity [26]. Adaptive immunity was also impaired by hyperglycemia, which increased the risk of infections and mortality. Therefore, tight glycemic control during hyperglycemic conditions was beneficial to the maintenance of the CMI in the host [27]. Metformin and sulphonylureas are still the first-line oral agents used for hyperglycemia in patients with type 2 diabetes mellitus because they are inexpensive and have fewer adverse effects. It is widely understood that many commonly used anti-diabetic agents exert glucose-lowering effects by different mechanisms of action on glucose metabolism and pancreatic insulin secretion. However, accumulating evidence has demonstrated that these anti-diabetic agents can exert significant anti-inflammatory effects to diminish overall diabetes-related complications, which are independent to its glucose-lowering efficacy. Thiazolidinediones (TZDs), metformin and insulin have demonstrated greater anti-inflammatory effects than sulphonylureas and alpha-glucosidase inhibitors [28]. Compared with TZDs or insulin, which increased the risk of HZ, the present study showed that metformin and sulphonylureas did not increase the risk of HZ in diabetic patients. The corollary to this notion is that patients given monotherapy such as metformin or sulphonylureas for hyperglycemia may be at the initial stage of diabetes in which their symptoms are relatively mild with minimal abnormal biochemical changes. However, when targeted monotherapy fails to yield a better glycemic control during the progression of the disease, combination therapy with stronger hypoglycemic agents is highly recommended. Therefore, we need to conduct further studies to determine whether tight glycemic control can be attained in our diabetic patients treated with anti-diabetic agents.

## Limitations

The main limitation of the present study is that the Taiwan NHIRD does not provide temporal blood glucose levels, HbA1c levels or dosages of anti-diabetic drugs. The lack of this data may have led to underestimation of the number of patients with mild symptoms who did not seek medical treatment, although such underestimation should remain stable over time.

## Conclusions

In summary, the present study demonstrated that there was an escalated risk of HZ occurrence in diabetic patients comorbid with coronary artery disease and associated microvascular disorders. In addition, a higher risk of HZ was significantly demonstrated in diabetic patients who were given insulin alone or in combination with other anti-diabetic agents compared to those who received monotherapy. Further studies with cohort design are necessary to clarify the effect of vaccination on the risk of developing herpes zoster in these groups of patients in Taiwan.
